# Role of albumin in the preservation of endothelial glycocalyx integrity and the microcirculation: a review

**DOI:** 10.1186/s13613-020-00697-1

**Published:** 2020-06-22

**Authors:** Cesar Aldecoa, Juan V. Llau, Xavier Nuvials, Antonio Artigas

**Affiliations:** 1 Department of Anaesthesiology and Surgical Critical Care, Hospital Universitario Rio Hortega, c/Dulzaina 2, 47012 Valladolid, Spain; 2 Department of Anaesthesiology and Surgical Critical Care, Hospital Universitario Dr. Peset, Universitat de València, c/Gaspar Aguilar 90, 46017 Valencia, Spain; 3 Intensive Care Unit, and SODIR Research Group, Vall d’Hebron Institut de Recerca (VHIR), Hospital Universitari Vall d’Hebron, Universitat Autònoma de Barcelona, Passeig Vall d’Hebron 119-129, 08035 Barcelona, Spain; 4 Critical Care Center, Corporacion Sanitaria Universitaria Parc Tauli, CIBER Enfermedades Respiratorias, Autonomous University of Barcelona, Parc Tauli 1, 08028 Sabadell, Spain

**Keywords:** Endothelial cell, Glycocalyx, Albumin, Microcirculation, Sepsis

## Abstract

The endothelial glycocalyx comprises a complex layer of membrane-bound proteoglycans, secreted glycosaminoglycans, glycoproteins, glycolipids and bound plasma proteins such as albumin and antithrombin associated with the endothelial surface. The glycocalyx plays an important role in vascular homeostasis, regulating vascular permeability and cell adhesion, and acts as a mechanosensor for hemodynamic shear stresses; it also has antithrombotic and anti-inflammatory functions. Plasma proteins such as albumin are physiologically bound within the glycocalyx, thus contributing to stability of the layer. Albumin is the major determinant of plasma colloid osmotic pressure. In addition, albumin transports sphingosine-1-phosphate which has protective endothelial effects, acts as a free radical scavenger, and has immunomodulatory and anti-inflammatory effects. This review examines the physiological function of the endothelial glycocalyx and the role of human albumin in preserving glycocalyx integrity and the microcirculation.

## The endothelial glycocalyx

### Composition and structure of the endothelial glycocalyx

**Fig. 1:**
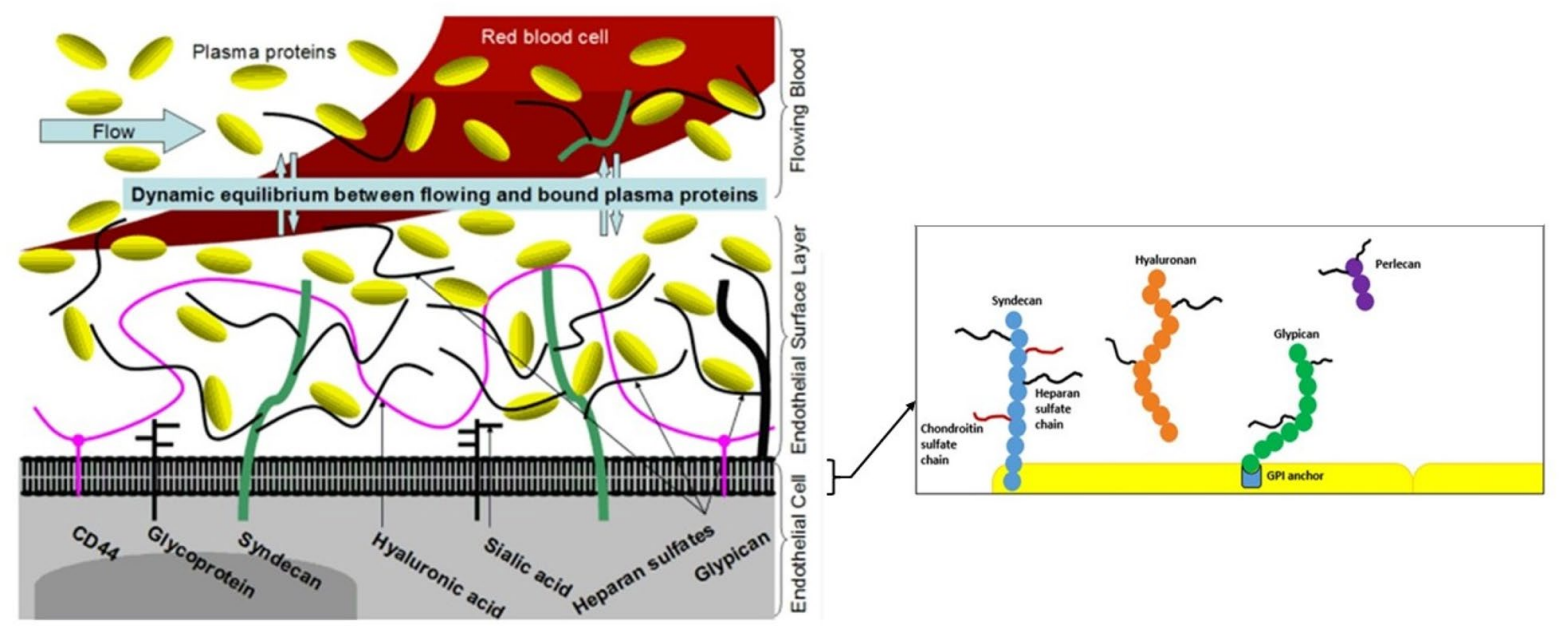
Structure of the endothelial glycocalyx illustrating proteoglycans and glycosaminoglycans. Reproduced with permission from [4]. *GPI* glycosylphosphatidylinositol

The endothelial glycocalyx is a complex carbohydrate-rich gel-like layer lining the luminal surface of blood vessels [1]; it functions as a barrier between the blood and vessel wall [2, 3]. The glycocalyx layer is composed of membrane-bound proteoglycans, secreted glycosaminoglycans (GAGs), sialic acid-containing glycoproteins, and glycolipids associated with the endothelial surface (Fig. 1) [4]. The main proteoglycans of the endothelial glycocalyx are membrane-spanning syndecans and glycosylphosphatidylinositol-linked glypicans which carry the two main GAGs, heparan sulfate and chondroitin sulfate, through covalent attachment to the protein core. Syndecans carry both GAGs, while glypicans carry only heparan sulfate. Table 1 summarizes the characteristics of core proteoglycans in the glycocalyx [5]. A third major GAG, hyaluronan, is secreted by endothelial cells but is not covalently linked to a core protein; it binds to cell surface adhesion receptors such as CD44. Plasma proteins such as albumin and antithrombin are also bound within the glycocalyx [4, 6–9].

**Table 1:** Characterization of proteoglycan core proteins in the glycocalyx. Adapted from [5]

Core protein	Core size (kDa)	Number of subtypes	Structural characteristics	Linked glycosaminoglycan (GAG)
Syndecan	19–35	4	Transmembrane protein	Heparan sulfate, chondroitin sulfate
Glypican	57–69	6	GPI-anchored protein	Heparan sulfate, chondroitin sulfate
Perlecan	400	1	Secreted	Heparan sulfate, chondroitin sulfate
Versican	370	1	Secreted	Chondroitin sulfate, dermatan sulfate
Decorin	40	1	Secreted	Chondroitin sulfate, dermatan sulfate
Biglycan	40	1	Secreted	Chondroitin sulfate, dermatan sulfate
Minecan	35	1	Secreted	Keratan sulfate

*GPI* glycosylphosphatidylinositol

**Fig. 2:**
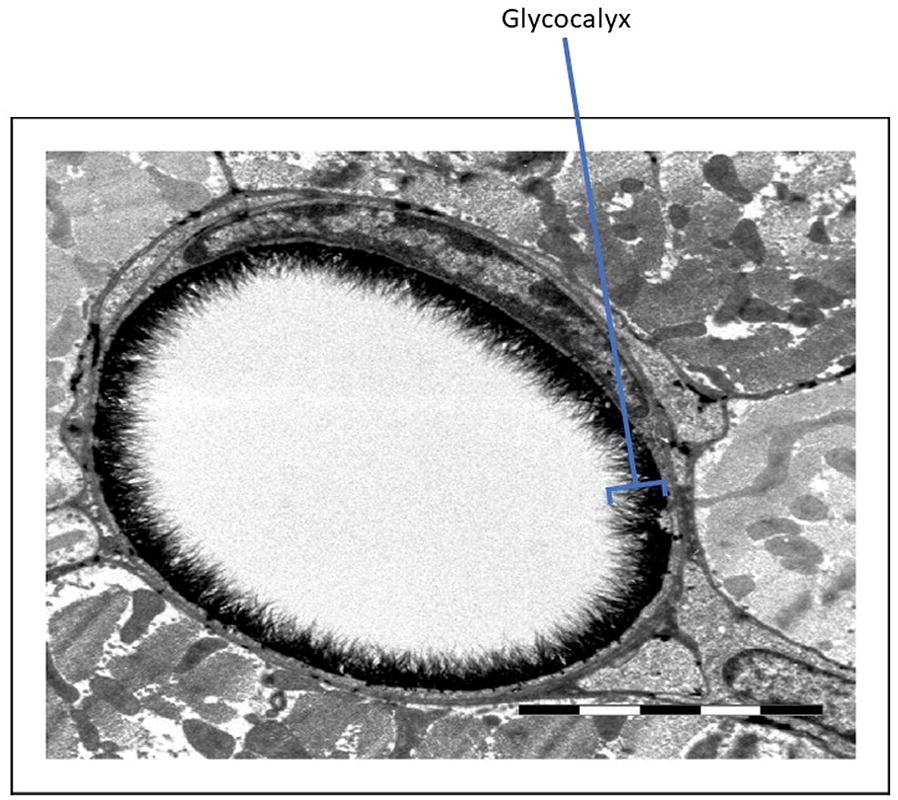
Electron micrograph of a cross-sectional image of a coronary endothelial glycocalyx (courtesy of B. van den Berg, Maastricht University). Reproduced with permission from [11]

The term endothelial surface layer is sometimes used to describe the intimal surface of blood vessels comprising the endothelial glycocalyx and associated components derived from endothelial cells and plasma [3, 10]. The thickness of the glycocalyx/endothelial surface layer (Fig. 2) [11] varies depending on the method used for measurement [9]. Intravital microscopy and orthogonal polarization spectral imaging are techniques which indirectly measure the endothelial glycocalyx in vivo, and the glycocalyx can be measured indirectly ex vivo using microparticle image velocimetry. The glycocalyx can be measured directly in vitro using transmission electron microscopy, confocal laser scanning microscopy and atomic force microscopy, and ex vivo using two-photon laser scanning microscopy [12, 13]. In humans, the mean microvascular glycocalyx thickness estimated using orthogonal polarization spectral imaging was about 0.5 µm (range 0.3 to 0.75 µm) [14].

### Physiological function of the endothelial glycocalyx

The outermost layer of the microvasculature serves as a regulatory barrier of vascular permeability. It participates in mechanotransduction by sensing fluid shear forces and regulating the vascular tone. The endothelial glycocalyx, which has an important role in maintaining vascular homeostasis [9], also has several anti-adhesive and antithrombotic effects on the surface of endothelial cells and can protect endothelial cells from oxidative stress [3, 6–9, 15, 16].

#### Regulation of vascular permeability and barrier function

**Fig. 3:**
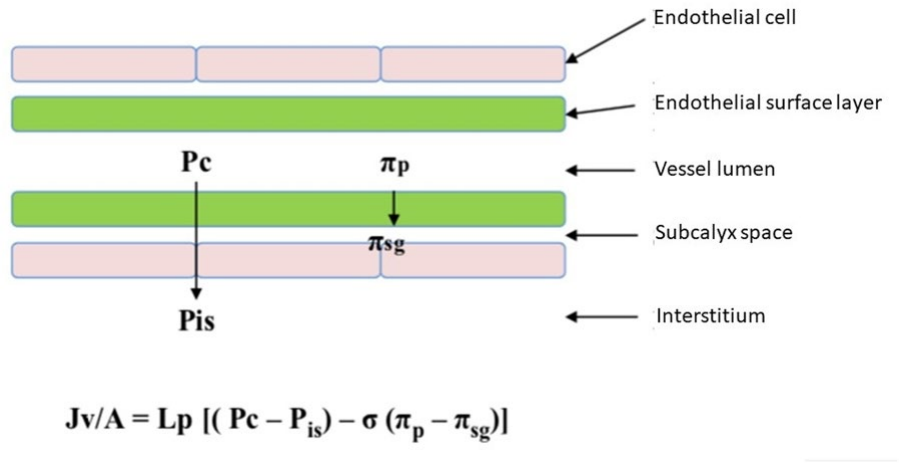
The revised Starling principle. Reproduced with permission from [2]. *Jv/A* filtered volume per unit area; *Lp* hydraulic conductance; *Pc* capillary hydrostatic pressure; *P*_*is*_ interstitial hydrostatic pressure; *σ* osmotic reflection co-efficient; *π*_*p*_ oncotic pressure in the luminal side of endothelial surface layer; *π*_*sg*_ oncotic pressure in the subglycocalyx space

Starling’s original model of transvascular fluid exchange, which depends on a balance between hydrostatic and oncotic pressure gradients in semi-permeable capillaries, fails to explain the clinical responses observed after fluid resuscitation. A revised Starling model, first proposed by Levick and Michel [17] which incorporates the effect of the endothelial glycocalyx, basement membrane and extracellular matrix on fluid exchange, provides a better explanation of fluid transvascular interchanges and a patient’s response to fluid resuscitation because the effect of the glycocalyx in reducing fluid extravasation was unknown. Consequently, in Starling’s original model, the observed extracellular volume distribution following fluid resuscitation was not predicted by the model [2, 16–19]. Starling’s original principle and the revised Starling equation and glycocalyx model are compared in Additional file 1: Table S1 [18] and shown in Fig. 3 [2]. The revised Starling model proposes that the endothelial glycocalyx is the key determinant of hydrostatic and oncotic pressure gradients between the capillary lumen and the interstitium. Important Starling forces are the transendothelial pressure difference (Pc − P_is_) and colloid osmotic pressure difference between plasma and the subglycocalyx (π_p_ − π_sg_). This oncotic pressure difference explains the failure of the interstitial protein concentration to influence fluid movement [2, 17, 18].

Under physiological conditions, the glycocalyx acts as a barrier against the shift of albumin and other circulating plasma components (mainly other proteins) across the endothelium [20, 21].

#### Mechanosensory function

Mechanical forces on endothelial cells generated by blood flow evoke biochemical responses that modulate endothelial structure and function through a process known as mechanotransduction. The glycocalyx acts as a cytoskeleton for endothelial cells. Mechanical distortion of ‘bush-like’ clusters of proteoglycans projecting from anchor points in the endothelial cell cytoskeleton generates forces which can deform the cytoskeleton. An associated increase in the expression of endothelial nitric oxide (NO) synthase catalyzes the production of NO, dilating vessels and reducing stress [2, 22, 23].

#### Rheological function

The glycocalyx has a vasculo-protective role by repelling red blood cells and by physically inhibiting the interaction of endothelial cell adhesion molecules (e.g., integrins and members of the immunoglobulin superfamily) with circulating platelets and leukocytes [9, 10].

#### Anticoagulation function

Several important anticoagulant mediators bind to GAGs located in the glycocalyx. These include antithrombin which binds via heparan sulfate to inhibit thrombin and activated factors IX and X; heparin cofactor II which is activated by dermatan sulfate; and tissue factor pathway inhibitor which binds via heparan sulfate to inhibit factors VIIa and Xa. Thrombomodulin, an endogenous anticoagulant produced by endothelial cells, interacts with thrombin to activate the protein C anticoagulant pathway [2].

#### Protective function against free radicals

Glycocalyx binding of enzymes such as extracellular superoxide dismutase protects endothelial cells against oxidative stress from reactive oxygen species (ROS) while maintaining NO availability, thus preventing endothelial dysfunction [9, 24].

### Physiological role of endogenous albumin

Although albumin has a net negative charge, its amphoteric nature promotes tight binding to the glycocalyx with the net effect of reducing hydraulic conductivity across the vascular barrier, resisting glycocalyx degradation (i.e., protecting against shedding) and thereby contributing to maintenance of vascular integrity and normal capillary permeability, and facilitating transmission of shear stress [2, 15, 23, 25].

Under physiological conditions, the concentration of intravascular albumin is the major determinant of plasma colloid osmotic pressure [18].

Exposed thiol groups on the albumin molecule act as a scavenger for ROS such as superoxide (O_2_^−^) and hydroxyl (^•^OH) radicals and reactive nitrogen species, e.g., peroxynitrite radicals. Albumin has an additional anti-oxidant effect through binding to free copper ions (Cu^2+^) which are known to accelerate the production of free radicals [26, 27].

Albumin also has immunomodulatory and anti-inflammatory effects through binding of bacterial products, modulation of antigen-presenting cell function, modulation of cytokine production, and reducing hypoxia-inducible factor-1α gene expression which is upregulated in response to low oxygen concentrations [25].

Along with lipoproteins, albumin has an important role in delivering sphingosine-1-phosphate (S1P) to the endothelial cell surface where it functions in maintaining normal vascular permeability [28]. S1P protects endothelial cells by suppressing the activity of metalloproteinases, stabilizes the glycocalyx by reducing GAG degradation and shedding [21, 22], and regulates barrier function by modulating the expression of vascular endothelial-cadherin and β-catenin at endothelial cell–cell contact regions [29].

Post-translational modifications of human serum albumin include glycation, cysteinylation, S-nitrosylation, S-guanylation and S-transnitrosation which can affect the binding of some exogenous drugs [30]. In addition, advanced glycation end (AGE)-modified albumin can induce proinflammatory signaling through activation of AGE receptors [31]. This was illustrated in a murine model of peritonitis and sepsis where administration of therapeutic infusion solutions containing high concentrations of AGE-modified albumin reduced survival [32].

## Alterations of the endothelial glycocalyx

### Pathologies/interventions associated with glycocalyx alterations

**Fig. 4:**
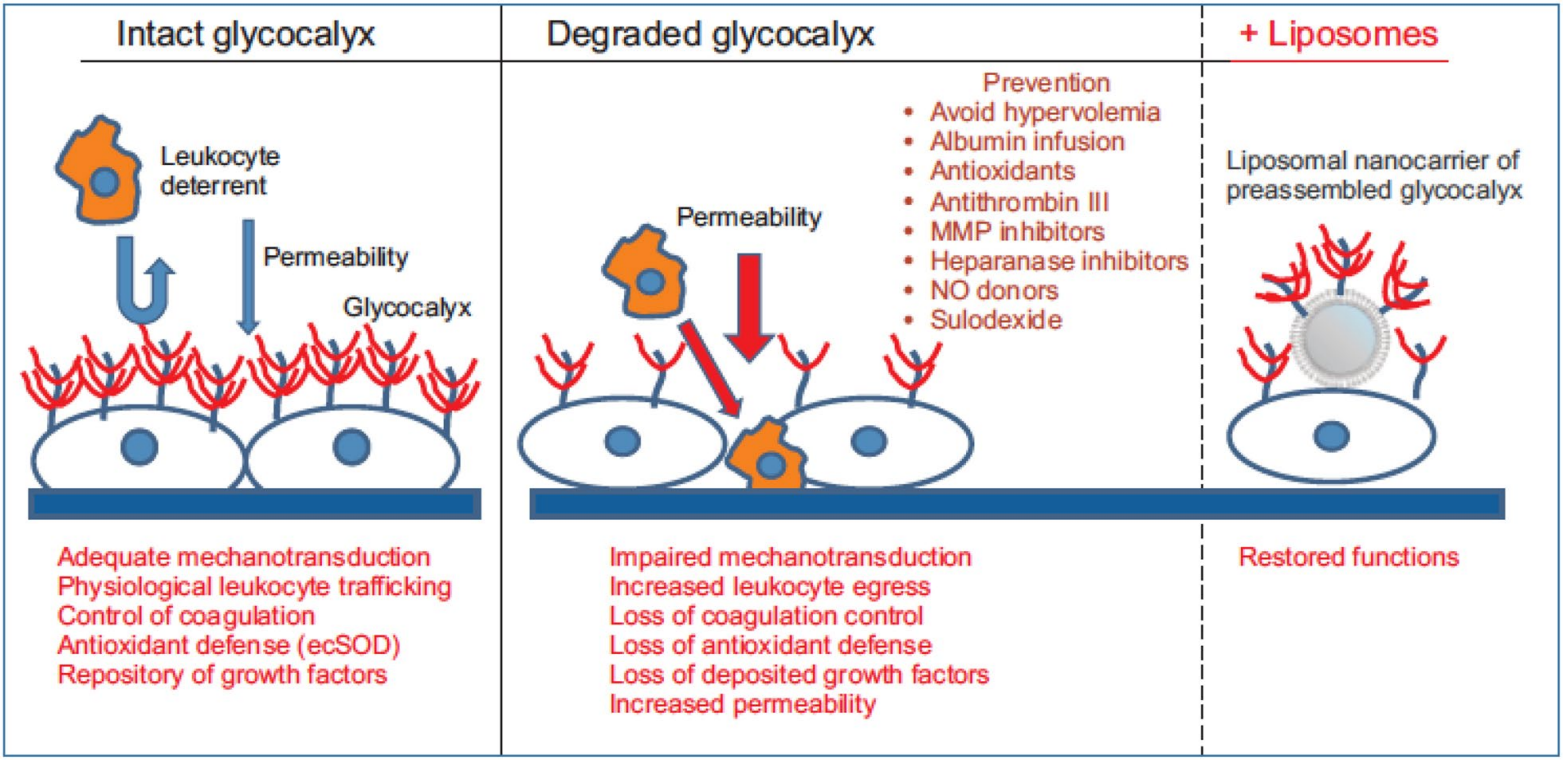
Pathologies/interventions associated with glycocalyx alterations. Reproduced with permission from [10]. *ecSOD* extracellular superoxide dismutase; *MMP* matrix metalloprotease; *NO* nitric oxide. Sulodexide is a highly purified glycosaminoglycan mixture of low molecular weight heparin plus dermatan sulfate [30]

Glycocalyx and endothelial cell damage, or endotheliopathy as it is known [33], occur in several clinical situations including ischemia–reperfusion injury, hypoxia/reoxygenation, inflammation, sepsis, hemorrhagic shock, hypervolemia, hyperglycemia, excessive shear stress and coronary artery bypass surgery [23, 34]. These injuries determine pathological changes in the endothelial glycocalyx such as impaired mechanotransduction, increased egress of leukocytes, loss of coagulation control, loss of anti-oxidant defense, loss of deposited growth factors, and increased vascular permeability (Fig. 4) [10, 35]. In a clinical context, disruption of the endothelial glycocalyx layer can lead to development of interstitial edema in some patients, notably those with inflammatory conditions such as sepsis [36].

#### Ischemia–reperfusion injury

Ischemia–reperfusion injury results in tissue damage following disruption of the glycocalyx [37]. Microvascular endothelial cell dysfunction produces organ dysfunction locally or systemically, including systemic inflammatory response syndrome [38]. Cardiac ischemia–reperfusion injury may occur during procedures such as coronary artery bypass grafting, percutaneous coronary angioplasty, and heart transplant surgery [39], with cardiac surgery per se also producing glycocalyx damage [40].

#### Sepsis

Glycocalyx shedding is a feature of inflammation, trauma and sepsis, and is mediated by pro-inflammatory agents including tumor necrosis factor-α, C-reactive protein, adenosine, bradykinin, histamine, platelet-activating factor, and bacterial lipopolysaccharide. Glycocalyx shedding leads to increased vascular permeability, tissue edema and relative hypovolemia [41–43].

Many features of endothelial dysfunction in sepsis are shared with aging and with numerous chronic diseases such as hypertension, dyslipidemia, diabetes mellitus (DM), cardiovascular disease (CVD), cerebrovascular disease, chronic kidney disease (CKD), chronic pulmonary disease, liver disease, and cancer. Common features include glycocalyx degradation and shedding; increased oxidative stress and systemic inflammation; intercellular junction disassembly, endothelial cell death and blood–tissue barrier disruption; enhanced leukocyte adhesion and extravasation; and induction of procoagulant and antifibrinolytic pathways [43].

#### Hemorrhagic shock

Endothelial glycocalyx shedding has been shown in rodent models of hemorrhagic shock, although the underlying mechanism is unknown [44–46]. Recent results in rats subjected to non-traumatic hemorrhagic shock showed glycocalyx degradation, which was independent of increased vascular barrier permeability [46].

#### Hyperglycemia

Evidence from rodent models [47] and clinical studies in volunteers [48] suggests that acute and chronic hyperglycemia can cause glycocalyx damage. The link between DM and CVD is well established with CVD being the most common cause of morbidity and mortality in diabetic patients [49]. A role for heparan sulfates in the development of widespread vascular endothelial damage leading to albuminuria and associated complications in patients with type 1 DM was suggested by Deckert and colleagues who formulated the ‘Steno hypothesis’ which proposes that albumin leakage results from extensive vascular damage [50]. Exposure of vascular endothelial cells to hyperglycemia and advanced glycosylation end products causes glycocalyx disintegration with increased leukocyte egress and release of human protease activated receptor 2 agonists, together with endothelial NO synthase uncoupling, resulting in reduced NO availability and increased vascular permeability [51].

#### Other

High-density lipoprotein cholesterol (HDL-C) may have a role as a causal contributor to sepsis survival [52, 53]. Low HDL-C levels have been shown to be a strong predictor of organ dysfunction or death in patients with suspected sepsis [54]. As HDL-C is able to bind and sequester pathogenic lipids (e.g., endotoxins), its modification might be a novel therapeutic strategy for treating sepsis [55].

### Potential effects of fluid therapy

#### Hypervolemia and type of fluid administered

A direct comparison of the hemodynamic effects of resuscitation fluids showed that colloids significantly increased plasma volume, cardiac index, and central venous pressure compared with crystalloids (*p* < 0.05), despite infusion of a higher volume of crystalloids (median 1800 vs 1500 mL) [55]. This is relevant because the duration of fluid infusion in fluid challenge significantly influences fluid responsiveness [56]. Fluid responsiveness does not equate with fluid requirement. Excess volume in the circulation is compensated by redistribution between stressed and unstressed volume [57] and, in the worst cases, by a leak to the interstitial space worsening tissue oxygenation [58]. A recently reported randomized trial found no differences between a slow (180 min) or rapid (30 min) infusion of 5% albumin on plasma volume expansion in patients following major abdominal surgery, and the rapid infusion had no effect on vascular leak [59].

As the type of fluid infused has an important effect on the glycocalyx, differences between albumin and crystalloids on glycocalyx function have been described.

In in vivo experiments of anesthetized rats subjected to hemorrhagic shock followed by fluid resuscitation, administration of normal saline failed to restore endothelial glycocalyx thickness and plasma levels of syndecan-1 (indicating failure to repair the glycocalyx), leading to a marked increase in vascular permeability and leukocyte rolling/adhesion. In contrast, albumin stabilized permeability and leukocyte rolling/adhesion, effects which were comparable to fresh frozen plasma. Albumin partially restored endothelial glycocalyx thickness, and lowered plasma syndecan-1 to baseline levels [60].

A recent review of commonly used resuscitation fluids for use in the critically ill highlighted the need to consider not only the oncotic properties of a fluid, but also its relative ability to protect and restore the endothelial glycocalyx. In this regard, evidence from observational and pre-clinical in vitro and in vivo studies indicates that albumin and fresh frozen plasma are superior to crystalloids and artificial colloids [61], although prospective studies are needed to confirm these findings.

### Markers of endothelial damage

Biomarkers of endothelial damage have been developed with most applied prognostically for conditions of systemic inflammation and sepsis [43, 62–71] (Box 1). In patients with septic shock, increased plasma angiopoietin-2 levels were associated with higher fluid overload, hepatic and coagulation dysfunction, acute kidney injury, mortality, and plasma cytokines, likely as the result of increased vascular leakage [72].

Assessment of glycocalyx damage using a variety of biomarkers has provided evidence of glycocalyx degradation in a range of clinical conditions including trauma, CKD, myeloid leukemia, acute decompensated heart failure, and Crohn’s disease [5].

Box 1. Biomarkers of endothelial damage

**Table:** 

Function	Biomarker	Reference
Markers of endothelial glycocalyx degradation	Urinary GAGs (heparan sulfate, chondroitin sulfate, hyaluronan and syndecan)	[62]
Markers of endothelial cell activation	Endocan	[63]
	Angiopoietin-1	[64–66]
	Angiopoietin-2	[64–68]
Cell adhesion molecules	Selectins	[68]
	Intercellular adhesion molecule 1 (ICAM-1); vascular cell adhesion molecule 1 (VCAM-1)	[63]
Vasoactive peptides	Mid-regional pro-adrenomedullin	[69]
	Mid-regional pro-ANP	[70]
Natural anticoagulants	Thrombomodulin	[70]
Polypeptides with vasoconstrictor and vasopressor activity	Endothelin	[68]
Growth factors	Vascular endothelial growth factor (VEGF)	[68]
Vascular damage	Circulating endothelial cells	[71]

*ANP* atrial natriuretic peptide, *GAGs* glycosaminoglycans

## Role of human albumin in maintaining glycocalyx integrity

### Pre-clinical studies of the effect of human albumin on the glycocalyx and microcirculation

Pre-clinical studies which illustrate the mechanism of action of albumin, and its effects in models of hemorrhagic shock, endotoxemia, vascular permeability and ischemia are summarized in Table 2 [13, 14, 60, 73–87]. Results from in vitro, in vivo, and ex vivo experiments illustrate the multifunctional nature of albumin including maintaining glycocalyx integrity and partially restoring impaired vascular permeability via release of S1P from RBCs; anti-inflammatory and anti-oxidative effects; improvement of the microcirculation and hemodynamics following hemorrhagic shock or endotoxemia; and acting as an effective plasma volume expander.

**Table 2:** Preclinical models of albumin

Preclinical model	Main findings	Reference
*Mechanism of action (MoA)*		
Rat perfused venular microvessel	Primary MoA of albumin in maintaining vascular permeability is release of S1P from RBCs	[73]
Rat experimentally induced hypovolemic shock	Albumin infusion partially restored the measured thickness of the endothelial glycocalyx and restored microvascular permeability. Restored permeability may be due to delivery of S1P to the endothelium and not wholly dependent on glycocalyx recovery	[74]
In vitro human uterine vein endothelial cells exposed to LPS and TNF-α	Human serum albumin (4%) inhibited inflammatory and oxidative stress pathways induced by endotoxins	[75]
In vitro model of inflammatory vascular injury using bovine aortic endothelial cells	Human serum albumin had modest intrinsic non-thiol-dependent anti-inflammatory effects	[76]
In vitro artificial semipermeable membrane	Albumin decreased water permeability of ultrafiltration membranes in a concentration dependent manner. Effects were mediated by plugging of the capillary pore and solute–solvent exchange at the capillary membrane surface	[77]
Atomic force microscopy and reflectance interference contrast microscopy of bovine lung endothelial cells	Albumin (0.1% and 4%) increased the thickness and produced softening of the glycocalyx compared with 1% albumin. Albumin produced glycocalyx softening in a concentration-dependent manner	[13]
*Models of hemorrhagic shock*		
Anesthetized rats subjected to hemorrhagic shock	Albumin partially restored endothelial glycocalyx thickness and stabilized permeability and leukocyte rolling/adhesion	[60]
Awake hamsters subjected to hemorrhagic shock	Albumin improved the microcirculation in correcting metabolic disorders (improving arterial base excess and oxygen extraction ratio) more effectively than RBC infusion	[78]
Rat intravital microscopy of the mesenteric microcirculation	Albumin improved microcirculation and global hemodynamics following hemorrhagic shock and attenuated the inflammatory response to reperfusion	[79]
*Models of endotoxemia*		
Mouse experimentally induced endotoxemia	Human serum albumin (4%) increased survival of endotoxemic mice compared with saline	[75]
Rat experimentally induced endotoxemia	Human serum albumin (4% or 20%) increased perfused vessel density and blood flow velocity and decreased flow heterogeneity to control values	[80]
Rat experimentally induced endotoxemia	Albumin (20%) improved hemodynamic parameters and microcirculatory perfusion; association with recovery of some glycocalyx components	[81]
*Models of vascular permeability*		
Ex vivo perfused isolated guinea pig heart	HES infusion, but not albumin infusion, significantly decreased net coronary fluid filtration	[82]
Rat experimentally induced hemorrhage or sepsis	Following hemorrhage or cecal ligation and incision, plasma volumes after albumin or crystalloid infusions were similar	[83]
Ex vivo perfused isolated guinea pig heart	Glycocalyx integrity was maintained with 1% human albumin and crystalloid, but functional breakdown of the vascular barrier was observed with 0.5% albumin and crystalloid	[84]
Rat anaphylactic shock	Under conditions of increased microvascular permeability, albumin (5%) was the most effective plasma volume expander compared with gelatin (4%), HES (6%) or saline	[85]
*Models of ischemia*		
Ex vivo perfused isolated guinea pig heart	Albumin was more effective than HES or saline in preventing cardiac fluid extravasation with ischemia–reperfusion injury	[86]
Rat transient focal cerebral ischemia	Compared with saline, albumin reperfusion had a neuroprotective effect, significantly increasing arteriolar diameter and improving venular and capillary erythrocyte perfusion with increased erythrocyte flow velocity	[87]

*HES* hydroxyethyl starch; *LPS* lipopolysaccharide; *MoA* mechanism of action; *RBC* red blood cell; *S1P* sphingosine-1-phosphate; *TNF*-*α* tumor necrosis factor alpha

### Clinical studies on the effect of human albumin in the glycocalyx and microcirculation

A recent study of patients with septic shock (*n* = 30) reported that, compared with saline, albumin improved skin endothelial cell function, improving microcirculatory blood flow. These beneficial effects may be independent of the oncotic properties of albumin as neither cardiac output nor skin blood flow differed between albumin- and saline-treated patients [88].

## Conclusions and expert opinion

The endothelial glycocalyx plays an important role in regulating vascular permeability. Glycocalyx and endothelial cell damage occurs in several clinical situations including sepsis, hemorrhagic shock, hypervolemia, and hyperglycemia. Albumin is physiologically bound within the glycocalyx, protecting against shedding and contributing to the maintenance of vascular integrity and normal capillary permeability. Owing to these properties, albumin has the potential to improve outcomes in clinical scenarios characterized by damaged glycocalyx. Based on our review and interpretation of the available literature, we provide an opinion on the most suitable applications for albumin and highlight areas which require additional research.

### Monitoring the microcirculation and endothelial damage

Several techniques are available to monitor the microcirculation and endothelial damage. These include: intravital microscopy in in vivo animal models for visualization of vascular dynamic events such as microvascular permeability, vasotone and blood flow [89] or the glycocalyx [90, 91]; assessment of the microcirculation with potential to measure the glycocalyx in critically ill patients [92]; application of near-infrared spectroscopy to measure tissue oxygenation [93, 94]; measuring skin mottling over the anterior surface of the knee [94]; measuring microalbuminuria [95]; biomarkers of acute kidney injury [96]; measuring protein concentrations in alveolar fluid lavage; and hemostasis-related biomarkers, e.g., factor VIII, von Willebrand factor, International Normalized Ratio, partial thromboplastin time and platelet count.

Intravital microscopy using sidestream darkfield (SDF) imaging is a non-invasive method increasingly used to analyze the sublingual microcirculation. The technique visualizes erythrocytes within the microvasculature due to light emitted by a light emitting diode probe which is reflected by hemoglobin and detected by a SDF camera [97]. Total vessel density, perfused vessel density, proportion of perfused vessels and microvascular flow index are traditionally estimated by offline computer analysis although, more recently, point-of-care approaches using validated automatic software platforms have been described [98, 99]. SDF imaging detection of RBCs is used as a marker of microvascular perfusion, and measurement of the perfused boundary region (PBR) as an indirect marker for endothelial glycocalyx barrier dimensions. In a large study of overweight and obese individuals, the PBR and presence of RBCs in the microvascular circulation were markedly associated [100]. Hand-held intravital microscopy showed that sublingual microvascular blood flow alterations are common in patients with sepsis, with blood flow abnormality related to disease severity [92, 101]. Furthermore, sublingual microvascular glycocalyx is damaged in critically ill patients, especially those with sepsis [102, 103], but also after cardiac surgery with cardiopulmonary bypass [104, 105] and in emergency room and intensive care unit patients [106]. However, in patients with sepsis, there was no association of PBR and syndecan-1 values with established microcirculatory parameters [102], likely indicating that both alterations occur independently. These data should be treated with caution as the reproducibility of three sublingual microcirculation parameters (vascular density, RBC filling and PBR) estimated by SDF imaging is controversial and large studies are required to achieve statistically significant effects [107]. However, some studies have shown good reproducibility with the method if consecutive measurements are averaged [106, 108, 109]. The accuracy of in vivo glycocalyx measurement has been analyzed further with an in vitro approach using atomic force microscopy [102, 110]. Consensus European Society of Intensive Care Medicine guidelines provide 15 recommendations for acquisition and interpretation of microcirculatory images obtained with hand-held vital microscopes for assessment of the microcirculation in critically ill patients [92].

According to our clinical and scientific judgement, biomarkers of endothelial damage and/or evaluation of the sublingual microcirculation may have a role in identifying subgroups of patients at risk of morbidity and mortality.

We consider that albumin should be used in accepted indications in which it has a proven positive risk:benefit balance, mainly in septic patients (for initial resuscitation after adequate crystalloid infusion and hypoalbuminemic septic shock), for burn shock resuscitation and fluid maintenance, and in some cases of liver insufficiency. The benefits of albumin may relate to its ability to restore function to damaged glycocalyx, although further studies are required to confirm this relationship and identify thresholds for optimal benefit. The mechanism of action of these restorative effects also needs to be elucidated.

### Further research

As the lung and kidney are the organs most affected by septic shock, pre-clinical investigation of the effects of albumin on permeability disorders of these organs should be conducted. The development of non-invasive imaging-based analysis tools to assess changes in permeability, diameter, and blood flow of vessels in response to specific stimuli is beginning to benefit research in both pre-clinical and clinical areas. It is likely that patients’ response to albumin may differ depending on localization of the blood vessel under investigation.

Detailed studies of the effect of albumin on oxidative stress are required and could be assessed using in vitro models (e.g., cultures of human endothelial cells and glycocalyx components), as these are more reproducible than pre-clinical animal studies.

The main objective of clinical studies of albumin should not be to evaluate its effect on overall mortality, but rather on more specific endpoints such as organ dysfunction. Investigating the economic impact of albumin and its long-term consequences is also advised. Studies should accrue biological information, i.e., justification for the selected objectives. Smaller studies with homogeneous populations are preferred to large multicenter studies of patients with clinical heterogeneity.

In summary, additional research needs to be conducted to clarify the role of albumin as a protector or restorer of damaged glycocalyx, with the aim of identifying clinical applications.

## Supplementary information

**Additional file 1: Table S1.** Comparison of Starling’s original principle and the revised Starling equation and glycocalyx model. Adapted from [18].

## Data Availability

Not applicable.
