# Supplementary material for: Role of albumin in the preservation of endothelial glycocalyx integrity and the microcirculation: a review
Source: Ann Intensive Care. 2020 Jun 22;10:85. doi: 10.1186/s13613-020-00697-1 (PMC7310051; doi:10.1186/s13613-020-00697-1)
Supplement: Supplementary file 1 — Additional file 1: Table S1. Comparison of Starling’s original principle and the revised Starling equation and glycocalyx model. Adapted from [18]. [file 13613_2020_697_MOESM1_ESM.docx]

**Table S1. Comparison of Starling’s original principle and the revised Starling equation and glycocalyx model.** Adapted from [18].

| **Original Starling principle** | **Revised Starling equation and glycocalyx model** |
| --- | --- |
| Intravascular volume consists of plasma and cellular elements | Intravascular volume consists of glycocalyx volume, plasma volume, and red cell distribution volume |
| Capillaries separate plasma with high protein concentration from interstitial fluid with low protein concentration | Sinusoidal tissues (marrow, spleen, and liver) have discontinuous capillaries and their interstitial fluid is essentially part of the plasma volume  Open fenestrated capillaries produce the renal glomerular filtrate  Diaphragm fenestrated capillaries in specialized tissues can absorb interstitial fluid to plasma  Continuous capillaries exhibit ‘no absorption’  The endothelial glycocalyx layer is semi-permeable to anionic proteins and their concentration in the intercellular clefts below the glycocalyx is very low |
| Important Starling forces are the transendothelial pressure difference and the plasma–interstitial colloid osmotic pressure difference | Important Starling forces are the transendothelial pressure difference and the plasma–subglycocalyx colloid osmotic pressure difference. Interstitial fluid colloid osmotic pressure is not a direct determinant of filtered volume (J_v_) |
| Fluid is filtered from the arterial end of capillaries and absorbed from the venous end. A small proportion returns to the circulation as lymph | J_v_ is much less than predicted by Starling’s principle, and the major route for return to the circulation is as lymph |
| Raising plasma colloid osmotic pressure enhances absorption and shifts fluid from interstitial fluid to plasma | Raising plasma colloid osmotic pressure reduces J_v_ but does not cause absorption |
| At subnormal capillary pressure, net absorption increases plasma volume | At subnormal capillary pressure, J_v_ approaches zero. Auto transfusion is acute, transient, and limited to about 500 mL |
| At supranormal capillary pressure, net filtration increases interstitial fluid volume | At supranormal capillary pressure, when the colloid osmotic pressure difference is maximal, J_v_ is proportional to the transendothelial pressure difference |
| Infused colloid solution is distributed through the plasma volume, and infused isotonic salt solution through the extracellular volume | Infused colloid solution is initially distributed through the plasma volume, and infused isotonic salt solution through the intravascular volume  At supranormal capillary pressure, infusion of colloid solution preserves plasma colloid osmotic pressure, raises capillary pressure, and increases J_v_  At supranormal capillary pressure, infusion of isotonic salt solution also raises capillary pressure, but it lowers colloid osmotic pressure and so increases J_v_ more than the same colloid solution volume  At subnormal capillary pressure, infusion of colloid solution increases plasma volume and infusion of isotonic salt solution increases intravascular volume, but J_v_ remains close to zero in both cases |
